# Blastomycotic arthritis and osteomyelitis in childhood: a case series

**DOI:** 10.1186/1546-0096-10-S1-A101

**Published:** 2012-07-13

**Authors:** Rachel R Johnson, Sheetal S Vora

**Affiliations:** 1 Medical College of Wisconsin Affiliated Hospital, Wauwatosa, WI, USA; 2 Medical College of Wisconsin and the Children's Research Institute, Wauwatosa, WI, USA

## Purpose

Blastomycosis is caused by the fungus B. dermatitidis endemic around the Great Lakes, the southeast, and the south central United States. Blastomycosis infections in children account for only 3-10% of total reported cases. Disseminated blastomycosis can cause arthritis and osteomyelitis. Juxta-articular involvement may lead to osteomyelitis. We present the first known case series of blastomycotic arthritis and osteomyelitis as presenting symptoms and asymptomatic findings in a pediatric population.

## Methods

The medical records of 31 children diagnosed with culture proven blastomycosis between 2000 and 2010 were retrospectively reviewed. Items analyzed included patient demographics, clinical history and physical exam with an emphasis on bone and joint, radiographic findings, leukocyte count, method of diagnosis, and anti-fungal drug choice and length of treatment.

## Results

In total, 7 of 31(22.6%) patients diagnosed with B. dermatitidis infection had bone and/or joint involvement. Ages ranged from 6 to 17 years and all were male. Six of the seven (85.7%) patients were urban dwellers with no known exposure to a rural setting. Almost all patients presented with either bone pain (1) and/or arthralgia (5). One patient had asymptomatic joint involvement discovered on imaging. On exam, five had signs of arthritis including joint effusion, erythema and warmth. Four had associated cutaneous involvement and three had lung involvement. Leukocyte count had a median value of 9.7 x 10^3/ul (range from 4.4 - 15.4 x 10^3/ul). Two patients had oligoarthritis, (two and three joints) involved, respectively. Predominantly, large joints were affected including elbow, wrist, knee, and hip. Diagnosis of B. dermatitidis was made by skin culture in three patients and bone culture in four patients. Six of the patients received IV amphotericin B for 2 months followed by oral itraconozole. One patient only received oral itraconazole. None of the patients had recurrence of infection after treatment.

## Conclusion

Blastomycosis can present as isolated arthritis or osteomyelitis with or without typical skin and/or respiratory involvement. Monoarticular arthritis is seen but multiple joints can be involved. B. dermitiditis appears to be increasing in incidence in the urban setting.

## Disclosure

Rachel R. Johnson: None; Sheetal S. Vora: None.

